# Detecting ice artefacts in processed macromolecular diffraction data with machine learning

**DOI:** 10.1107/S205979832101202X

**Published:** 2022-01-21

**Authors:** Kristopher Nolte, Yunyun Gao, Sabrina Stäb, Philip Kollmannsberger, Andrea Thorn

**Affiliations:** a Institute for Nanostructure and Solid State Physics, University of Hamburg, Luruper Chaussee 149, 22761 Hamburg, Germany; b Center for Computational and Theoretical Biology, University of Wuerzburg, Campus Hubland Nord 32, 97074 Wuerzburg, Germany

**Keywords:** machine learning, convolutional neural networks, macromolecular crystallography, ice rings, *AUSPEX*

## Abstract

Contamination with diffraction from ice crystals can negatively affect, or even impede, macromolecular structure determination, and therefore detecting the resulting artefacts in diffraction data is crucial. However, once the data have been processed it can be very difficult to automatically recognize this problem. To address this, a set of convolutional neural networks named *Helcaraxe* has been developed which can detect ice-diffraction artefacts in processed diffraction data from macromolecular crystals. The networks outperform previous algorithms and will be available as part of the *AUSPEX* web server and the *CCP*4-distributed software.

## Abstract 2

A program utilizing artificial learning and convolutional neural networks, named *Helcaraxe*, has been developed which can detect ice-crystal artefacts in processed macromolecular diffraction data with unprecedented accuracy.

## 1. Introduction

**Figure 1:**
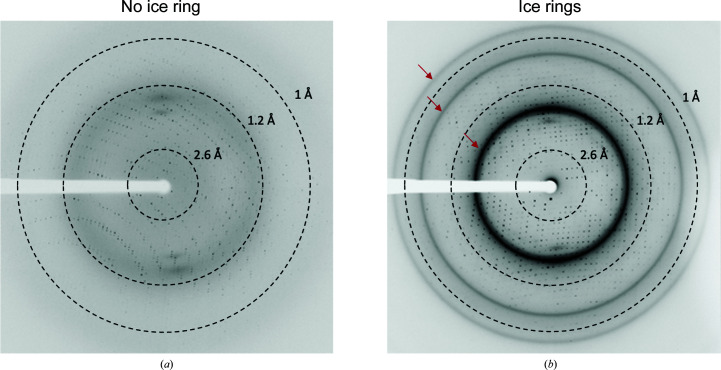
Diffraction patterns of dihydrofolate reductase from *Mycobacterium ulcerans*. The resolution is marked by dashed lines. (*a*) shows a pattern without ice rings (PDB entry 7k6c) and (*b*) shows a pattern with ice-crystal artefacts (indicated by the red arrows; PDB entry 7km9).

Crystals of biological macromolecules are routinely cryocooled to a temperature of 100 K before exposure to X-rays to reduce radiation damage during the diffraction experiment (Garman & Weik, 2019 ▸). Cryocooling can lead to the formation of ice (Garman & Owen, 2006 ▸). While antifreeze agents and flash-cooling are commonly employed to minimize this, rings from the diffraction of small ice crystals are frequently found in diffraction images from cryocooled macromolecular samples (Chapman & Somasundaram, 2010 ▸; Fig. 1 ▸). The emergence of fast-readout pixel detectors, which are ideally used to measure finely sliced diffraction images, makes the visual identification of ice artefacts from individual images more difficult (Thorn *et al.*, 2017 ▸). Ice rings can be observed when several images are averaged to increase the contrast or through newly developed machine-learning approaches (Czyzewski *et al.*, 2021 ▸).

Identifying whether a structure, or more exactly the integrated, scaled and merged diffraction data set, available in the worldwide Protein Data Bank (wwPDB; Berman *et al.*, 2000 ▸) is affected by ice-ring contamination is even more difficult. Only a few entries have the corresponding raw data (*i.e.* the images) available, as these are neither required for publication nor can be deposited directly in the wwPDB. However, if an integrated and merged data set is affected by ice diffraction, one can assume that subsequent model refinement will be affected. It has been demonstrated that removing ice rings from the data during integration improves the *R* values by as much as 4.8% (Parkhurst *et al.*, 2017 ▸). Thus, the correct identification of ice rings in data sets is an important step in assessing and ultimately improving data quality.

**Figure 2:**
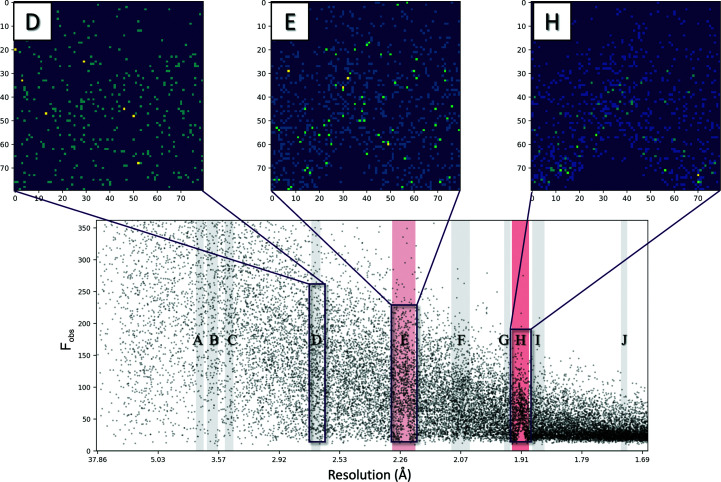
Relationship between the *AUSPEX* plot (bottom; PDB entry 4epz) and the *Helcaraxe* plots (top), which are 80 × 80 pixel histograms of the respective areas. *Helcaraxe* axis labels refer to pixel numbers. Ice-ring features were automatically identified by the statistical *AUSPEX* Icefinder score (shaded pink bars). The grey bars in the *AUSPEX *plot indicate the resolution ranges in which an ice ring can appear (letters A–H) that are used to generate the focused plots (top). The rectangles indicate the areas that the upper plots display. *Helcaraxe* plots E and H show ice-ring characteristics (spikes), while plot D shows none.

In addition to statistical identification in *CTRUNCATE* (Winn *et al.*, 2011 ▸) and *phenix.xtriage* (Adams *et al.*, 2010 ▸), the *AUSPEX* Icefinder score, recently improved by Moreau *et al.* (2021 ▸), is one of the most reliable statistical tools to detect ice-crystal artefacts in integrated, merged and scaled diffraction data sets. While statistical identification can identify stronger ice diffraction in processed data automatically, less distinct ice rings can go unnoticed. For this reason, in addition to automatic indication, *AUSPEX* also produces plots of observed intensities (*I*_obs_) (or structure-factor amplitudes *F*_obs_) against resolution, which permit the easy visual identification of ice-ring contamination as spikes (Fig. 2 ▸; Thorn *et al.*, 2017 ▸).

The discrepancy that humans can easily recognize ice rings in these *AUSPEX* plots while automatic statistical detection remains difficult led us to attempt identification using artificial intelligence. In recent years, the use of convolutional neural networks (CNNs) for data-driven research has enabled the identification and recognition of complicated patterns in noisy data (Schmidt *et al.*, 2019 ▸), leading to advances in all disciplines of science and data analysis. CNNs are exceptionally suited to the classification of multi-dimensional arrays because they can retain spatial input information (Yamashita *et al.*, 2018 ▸). Here, we present the results of employing CNNs to detect ice artefacts in processed macromolecular diffraction data.

## 2. Methods

### 2.1. Selection of training, validation and test data

1827 integrated, scaled and merged diffraction data sets indicated to have been measured at 100 K were used to generate training and validation sets (see supporting information). These diffraction data were randomly selected from the Coronavirus Structural Task Force repository (Croll *et al.*, 2021 ▸; 396 diffraction data sets), the Integrated Resource for Reproducibility in Macromolecular Crystallography (Grabowski *et al.*, 2016 ▸; 280 diffraction data sets) and the Protein Data Bank (Berman *et al.*, 2000 ▸; 1151 diffraction data sets) without duplicates. Diffraction data were used in MTZ format, obtained through the conversion of sf.cif files by *CIF*2*MTZ* from *CCP*4 (Winn *et al.*, 2011 ▸). If the CIF files had no observed intensities (*I*_obs_), structure-factor amplitudes (*F*_obs_) were used instead. If MAD data had been measured, the wavelength listed first in the deposited CIF file was used.

To convert the data into a format that could be presented to a neural network, two-dimensional histograms of *I*_obs_ or *F*_obs_ against resolution were generated, dubbed ‘*Helcaraxe* 1 plots’, using the NumPy *histogram*2*d* function (Virtanen *et al.*, 2020 ▸; example code is given in the supporting information). The size of the histograms was set to 80 × 80 pixels as this has proven to be the best compromise between information loss and data size. Multiple *Helcaraxe* plots were produced per data set, one around every expected ice-ring position present in the overall resolution range of the diffraction data set (Fig. 2 ▸, top). The width of individual ice-ring resolution ranges has previously been identified (Thorn *et al.*, 2017 ▸). To avoid extreme intensity outliers and for normalization, the lower limit of the *y* axis was set at the 0.5th percentile of intensities or amplitudes and the top limit at the 95th percentile. These parameters have proven to be the best middle ground between data loss and plot similarity. The *x* axis was scaled to obtain a constant histogram size despite the different widths of the individual ice-ring resolution ranges.

80% of the *Helcaraxe* plots generated from the MTZ files were allocated randomly to the training set and 20% to the validation set. The validation set was used to evaluate the CNNs during training and to select two final CNN candidates for the *Helcaraxe* program. The test set was not used in training.

A set of 200 randomly chosen diffraction data sets labelled for ice-ring contamination [previously published as Test Set C in the supporting information to Thorn *et al.* (2017 ▸) and reproduced here as supporting information] was assigned as a test set.

### 2.2. Data annotation

For annotation, ice rings were first identified in diffraction data sets using *AUSPEX* plots. Subsequently, *Helcaraxe* plots for the training, validation and test sets were generated as described and manually annotated for the presence of ice rings using the previous annotation results as guidance. A *Helcaraxe* plot was labelled as contaminated by ice diffraction if at least two of the following criteria were met.

(i) A vertical shift of the *I*_obs_ or *F*_obs_ values in the shape of a spike must be visible to the naked eye.
(ii) At least 1% of the area of the plot must be affected by the ice ring, meaning that either part of the plot was blank because of the ice ring or the intensities were shifted upwards. The area was measured by overlaying a grid.
(iii) The ice ring must be visible in the corresponding *AUSPEX* plot.

486 (26.6%) of the 1827 diffraction data sets used for the training and validation sets were found to have ice-diffraction contamination according to the aforementioned criteria. This resulted in the generation of 13 170 individual *Helcaraxe* plots, of which 984 (7.47%) were annotated as contaminated (see Table 1 ▸ and supporting information).

The test set (Test Set C in the supporting information to Thorn *et al.*, 2017 ▸), which was previously labelled for ice-ring contamination, includes ice-ring classification from other software and was also used by Moreau and coworkers to investigate the performance of their algorithm (Moreau *et al.*, 2021 ▸). The test set was again reviewed, consulting the annotation of Moreau and coworkers, and nine labels (4.5%) were updated (see supporting information). Three diffraction data sets were omitted because they had been superseded or had a lower maximum resolution than those ranges contaminated by ice rings. Of the 197 diffraction sets, 40 (20.3%) were labelled as containing an ice ring. There was no overlap between the training/validation set and the test set.

### 2.3. Network architecture and training

**Figure 3:**
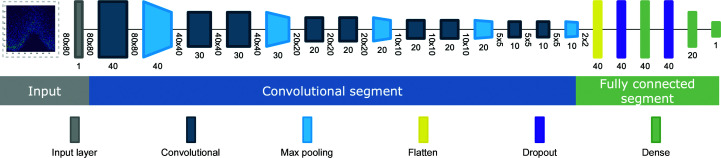
Schematic of the *Helcaraxe* network architecture. Input dimensions are shown as vertical numbers and the filter sizes are shown as horizontal numbers. Input is grey, convolutional layers are teal and pooling layers are light blue. Fully connected dropout layers are shown in purple and fully connected dense layers are shown in green. The image was created with *Net*2*Vis* (Bäuerle *et al.*, 2019 ▸).

**Figure 4:**
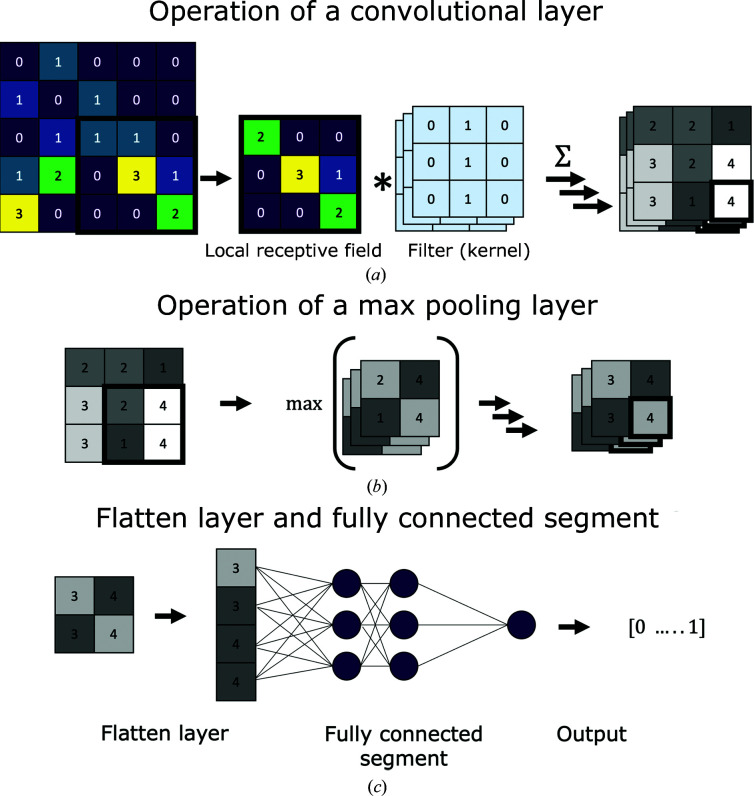
(*a*) General operation of a convolutional layer to create a feature map. Colours and grey shades refer to example numbers written in the fields. A patch (the local receptive field) of the input array is altered through multiple filters and the resulting values are saved in the feature map. (*b*) Like a convolutional layer, the pooling layer is only connected to a limited group of inputs within a rectangular field. However, it has no filter and only summarizes the input matrix through aggregation. (*c*) The flatten layer flattens the multidimensional matrix into a single-dimensional matrix; the output is passed to a regular neural network made of fully connected artificial neurons.

The network architecture of the employed CNNs consists of a convolutional and a fully connected part (Fig. 3 ▸). Two networks with the same architecture were trained, one for *I*_obs_ values and one for *F*_obs_ values. *Helcaraxe* plots were supplied through an input layer that passes the plot directly to the subsequent convolutional segment. The first segment of the network extracts data features and consists of four blocks, with each block having two convolutional layers followed by an aggregating max pooling layer (Fig. 4 ▸), with batch normalization layers in between to reduce the risk of overfitting through normalization. The second segment is connected through a flatten layer and contains two fully connected layers of artificial neurons separated by dropout layers which randomly omit neurons during training to further reduce the risk of overfitting (Srivastava *et al.*, 2014 ▸). The output layer is a single neuron that uses a sigmoid activation function. Therefore, a value (hereafter referred to as a prediction) between 0 (no ice-diffraction artefacts) and 1 (ice-diffraction artefacts) is returned for each *Helcaraxe* plot. The threshold for classification was 0.5.

The parameters which control the learning process (the hyperparameters) were optimized using the *Hyperband* optimization algorithm (Li *et al.*, 2018 ▸). The network used for predicting ice-diffraction *F*_obs_ plots (hereafter referred to as the *F*_obs_ network) was trained and validated only using *F*_obs_ *Helcaraxe* plots. The network for predicting *I*_obs_ plots (hereafter referred to as the *I*_obs_ network) was trained through transfer learning, fine-tuning the *F*_obs_ network using *I*_obs_ plots and a very moderate rate of learning (0.0005). This was performed to make sure that the network could adapt to the differences in the *I*_obs_ *Helcaraxe* plots without overriding the pattern-recognition abilities that had already been acquired by the *F*_obs_ network.

Network design and training were performed using *TensorFlow* 2.4.1 (Abadi *et al.*, 2016 ▸). The final trained networks were selected from multiple training runs based on their performance against the validation set. Their ability to operate reliably on unseen data was tested using the independent test set.

## 3. Results

### 3.1. Data features

**Figure 5:**
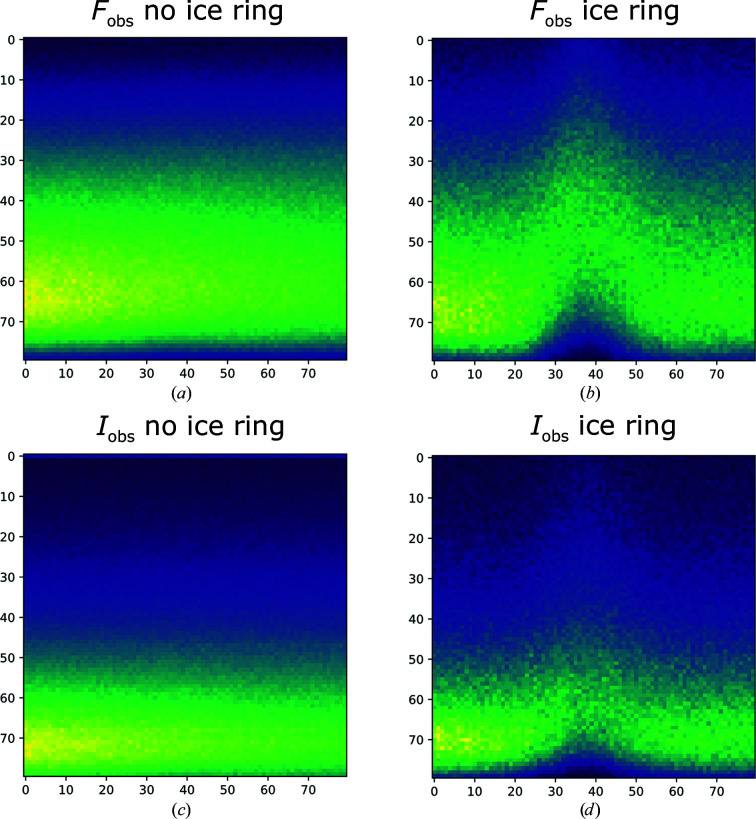
Averaged *Helcaraxe* plots of training and validation data, annotated as containing or not containing ice artefacts, for both *F*_obs_ and *I*_obs_. The characteristic ice spike can be seen in both mean ice-ring plots (*b*, *d*). The spike is more prominent for *F*_obs_. Axes refer to pixel values.

To acquire an overview of how ice-diffraction artefacts manifest in *Helcaraxe* plots, all plots from the training and validation sets which had been manually annotated as not containing ice diffraction were averaged for amplitudes and intensities, respectively (Figs. 5 ▸ *a* and 5 ▸ *c*), as were all plots annotated as containing ice diffraction (Figs. 5 ▸ *b* and 5 ▸ *d*). The resulting averaged plots of intensities or structure-factor amplitudes with no ice show a uniform vertical gradient. The corresponding plots with ice artefacts show an upward shift in the form of a spike in the middle. It is apparent that the points spread more evenly in *F*_obs_ plots than in *I*_obs_ plots. Spikes are also more visually prominent in Fig. 5 ▸(*b*) than in Fig. 5 ▸(*d*), which is potentially a consequence of the conversion of intensities into amplitudes by the French and Wilson method (French & Wilson, 1978 ▸), which imposes distribution expectations in order to facilitate conversion.

### 3.2. Network performance

Two trained networks were selected from multiple training runs based on their performance on the validation set. They were both evaluated against the test set (see Section 2.1) to confirm that they can generalize. The performance was measured using three metrics, accuracy (1), sensitivity (2) and specificity (3).

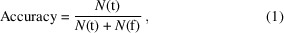

where *N*(t) is the number of true classifications and *N*(f) is the number of false classifications.

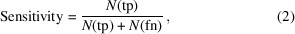

where *N*(tp) is the number of true positive classifications and *N*(fn) is the number of false negative classifications. 

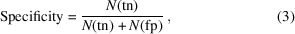

where *N*(tn) is the number of true negative classifications and *N*(fp) is the number of false positive classifications.

Judging by these criteria, the networks perform well on the validation set used in network training and the independent test set (see Table 2 ▸), showing that there was no overfitting of the networks with regard to the training set. The performance of both networks is sufficient to detect ice rings in most cases. Both networks have a higher specificity than sensitivity.

**Figure 6:**
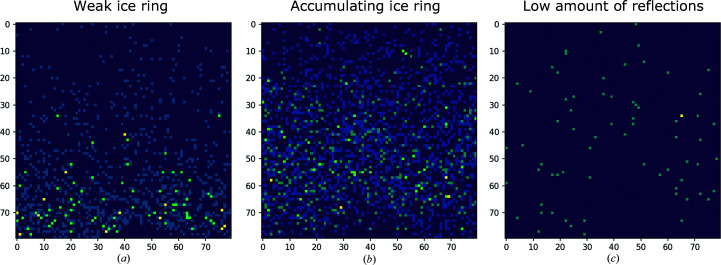
Misclassified *Helcaraxe* plots. Axis labels refer to pixels. Weak (*a*) and ‘accumulating’ (*b*) ice rings in plots were the main causes of false negative classifications. ‘Accumulating’ refers to ice rings which do not affect all intensities equally at a given resolution. Diffraction data with a low number of reflections (*c*) were a source of false positive misclassifications.

Closer visual inspection of the false negative classifications reveals that a portion of all test plots in the test set which were falsely identified as negative had only very small spikes affecting approximately 1% of the plot [as described in definition (ii) in Section 2.2] (*I*_obs_, seven of the 12 false negative classifications; Fig. 6 ▸ *a*) or the diffraction data set contained relatively few reflections (*I*_obs_, five of the 12 false negative classifications; *F*_obs_, two of the 13 false negative classifications; Fig. 6 ▸ *c*). Another cause of false negative classification is the presence of data points in the area of the ice spike (*I*_obs_, four of the 12 false negative classifications; *F*_obs_, eight of the 13 false negative classifications; Fig. 6 ▸ *c*). We suspect that this occurs when ice crystals build up during measurement so that both contaminated and uncontaminated intensities are present in the merged diffraction data. The main reason for false positive classification was a shift or absence of intensities in the usual ice-ring range without the typical shape of a spike [as described in definition (i) in Section 2.2] (*I*_obs_, 7 of the 13 false positive classifications; *F*_obs_, two of the four false positive classifications).

**Figure 7:**
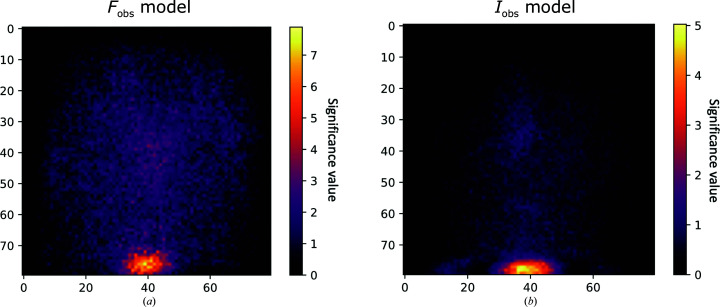
A *SmoothGrad* averaged sensitivity mask of the *F*_obs_ and *I*_obs_ networks against the test set, with pixel numbers indicated on the *y* and *x* axes. Larger values indicate a higher significance of the pixel. The area where ice artefacts usually appear has a higher relevance than the top, left and right edges. *F*_obs_ plots have a more spread-out distribution, which is likely to be the reason why the *F*_obs_ network also has a larger relevant area.

To obtain insight into the decision-making process of the networks, *SmoothGrad* (Smilkov *et al.*, 2017 ▸) was used to generate sensitivity maps that highlight which area has the most impact on the classification of the *Helcaraxe* plot. The area at the bottom, especially in the middle (where ice rings usually appear), had the greatest influence on the decision of the network. The edges at the top, right and left had close to no impact on the classification. Fig. 7 ▸ shows that the networks recognize the characteristic ice spike in *Helcaraxe* plots and use it as indicator for the classification. A comparison of the two sensitivity maps suggests that the two models have adapted to the properties (as described in Section 3.1) of their respective *Helcaraxe* plots (as described in Section 3.1). *F*_obs_ plots have a more spread-out distribution than *I*_obs_ plots and the sensitivity map shows that the *F*_obs_ model is sensitive to a broader area.

### 3.3. Comparison with other algorithms

The performance of both the *F*_obs_ and *I*_obs_ networks against the test set was compared with other ice-ring detection algorithms, namely *phenix.xtriage* (Adams *et al.*, 2010 ▸), *CTRUNCATE* (Winn *et al.*, 2011 ▸), the *AUSPEX* Icefinder score and the recent *p*_ice_ algorithm (Moreau *et al.*, 2021 ▸). *Helcaraxe* rates the individual resolution ranges in which ice rings can appear (as described in Section 3.1) and not the complete diffraction data set. Therefore, a data set was labelled as ice ring-contaminated when the network classified even a single *Helcaraxe* plot as contaminated.

The algorithm recently introduced by Moreau and coworkers outperformed all previous statistical methods. *Helcaraxe* reaches an even higher accuracy and sensitivity (both over 93%). Using *I*_obs_ plots *Helcaraxe* has a specificity of 92% and using *F*_obs_ plots it has a specificity of 97%. In general, the use of *Helcaraxe* results in the most reliable output in comparison to the traditional statistical methods (Table 3 ▸).

### 3.4. Analysis of the PDB

**Figure 8:**
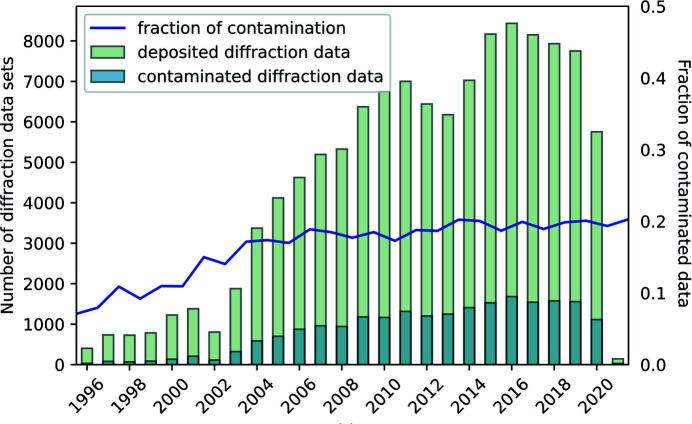
The number of deposited diffraction data sets (green) and the amount of these depositions which were annotated by *Helcaraxe* as containing at least one ice ring (blue). The contamination proportion is shown as a purple line.

The *Helcaraxe* networks were then used to analyse 117 615 randomly selected diffraction data sets deposited in the PDB (see Fig. 8 ▸). We found that 21 741 PDB entries (18.5%) show evidence of ice contamination in the processed, scaled and merged diffraction data. This number is similar to other previous large-scale analyses of the PDB (19% in Thorn *et al.*, 2017 ▸; 16% in Moreau *et al.*, 2021 ▸). We analysed the historical evolution of ice contamination. The presence of ice rings started increasing in the late 1990s when cryotechniques became routine at the first synchrotron MX beamlines and steadily grew when the first sample changers came online. Since the mid-2000s, the fraction of ice-contaminated data has stabilized at around 19%, even though the advent of pixel detectors has translated into shorter measurement times and consequently less time in which the protein crystal is exposed to a cryo-stream where it may accumulate surface ice crystals on the sample. The data produced in this analysis could be a useful starting point for research into the impact that ice rings have on structure solution and will be available from the *Helcaraxe* git repository (https://github.com/thorn-lab/helcaraxe).

### 3.5. Integration into *AUSPEX*

*Helcaraxe* has been integrated into a new Python-based version of *AUSPEX* (to be published) and can be used to automatically decide whether a specific resolution range contains ice-ring artefacts. The previous Icefinder score (Thorn *et al.*, 2017 ▸) is still used to rate the severity of the artefacts, as *Helcaraxe*, which is trained only for the detection and not the assessment of ice rings, provides a less differentiated classification. An additional discriminator function in the *Helcaraxe* script detects plots that are completely or partially blank, for example because resolution ranges were omitted during integration. This is achieved by checking, for specified resolution bins, whether the mean *I*_obs_ (or *F*_obs_) is close to 0. If this is true the plot is marked as nonpredictable and a warning is passed to the user. The runtime of *AUSPEX* is barely influenced by *Helcaraxe* as the prediction is fast (1–3 s per diffraction data set). No additional input for *AUSPEX* is needed to use *Helcaraxe*, and users of *AUSPEX* can choose between the *Helcaraxe* networks or *AUSPEX* Icefinder score to detect potential ice-crystal artefacts.

## 4. Conclusion

The identification of ice-diffraction artefacts in integrated, scaled and merged data has been an ongoing problem in macromolecular crystallography, even with modern cryocooling techniques (Moreau *et al.*, 2021 ▸) and new background-estimation algorithms (Parkhurst *et al.*, 2017 ▸). To aid identification in automatic pipelines as well as by users, a set of neural networks named *Helcaraxe* was developed to identify whether a scaled and merged X-ray diffraction data set contains ice-diffraction contamination of the reflection data from a macromolecular crystal. This program presents a significant improvement over previous automatic tools using classical statistical indicators. One area of future exploration would be to combine these approaches: reliable statistical methods such as those recently introduced by Moreau and coworkers could be used as an additional feature in the fully connected part of the *Helcaraxe* networks. Our work also shows that the multi-dimensional pattern-recognition abilities of convolutional neural networks are a valuable addition to the toolbox of diffraction data analysis, and the authors of this paper expect to see an increase in AI methods in this field in the near future. *Helcaraxe* is currently already in use in the Coronavirus Structural Task Force pipeline (Croll *et al.*, 2021 ▸) and has been integrated into the newest version of *AUSPEX*, which is available through the *AUSPEX* webserver (https://www.auspex.de).

## Supplementary Material

Performance results of Helcaraxe with the test set. DOI: 10.1107/S205979832101202X/gm5087sup1.xlsx

Annotation of training data. DOI: 10.1107/S205979832101202X/gm5087sup2.xlsx

AUSPEX ice-ring ranges. DOI: 10.1107/S205979832101202X/gm5087sup3.xlsx

Example code for Helcaraxe (zip archive). DOI: 10.1107/S205979832101202X/gm5087sup4.zip

## Figures and Tables

**Table 1:** Composition of the training, validation and test sets: numbers of *Helcaraxe* plots divided into *F*_obs_ and *I*_obs_ The percentages of plots with ice-ring contamination are given in parentheses.

	Training	Validation	Test
*F*_obs_	**6849**	**1713**	**1032**
No ice contamination	6251	1564	977
Ice contamination	598 (8.73%)	149 (8.70%)	65 (6.24%)
*I*_obs_	**3686**	**922**	**1410**
No ice contamination	3497	874	1329
Ice contamination	189 (5.13%)	48 (5.21%)	81 (5.74%)

**Table 2:** Performance of the final *F*_obs_ and *I*_obs_ networks against the *Helcaraxe* plots of the validation and test sets Accuracy, sensitivity and specificity values are given with a 95% confidence interval.

	True positive	False positive	True negative	False negative	Accuracy	Sensitivity	Specificity
*F*_obs_, validation	92	8	1556	57	0.962 ± 0.009	0.617 ± 0.003	0.995 ± 0.0001
*F*_obs_, test	42	4	973	13	0.984 ± 0.007	0.764 ± 0.004	0.996 ± 0.0001
*I*_obs_, validation	37	6	868	11	0.982 ± 0.009	0.771 ± 0.003	0.993 ± 0.0001
*I*_obs_, test	69	13	1316	12	0.982 ± 0.007	0.852 ± 0.002	0.990 ± 0.0002

**Table 3:** Comparison of different ice-detecting software *p*_ice_ is the new score introduced by Moreau and coworkers based on the previous *AUSPEX* Icefinder score. The data for accuracy, sensitivity and specificity are given with a 95% confidence interval.

Program	True positives	True negatives	False positive	False negatives	Accuracy	Sensitivity	Specificity
*phenix.xtriage*	13/40	144/157	13	27	0.80 ± 0.056	0.33 ± 0.005	0.92 ± 0.001
*CTRUNCATE*	22/40	93/157	64	18	0.58 ± 0.069	0.55 ± 0.005	0.59 ± 0.002
*AUSPEX*	23/40	141/157	16	17	0.83 ± 0.052	0.58 ± 0.005	0.90 ± 0.002
*p*_ice_	29/40	148/157	9	11	0.90 ± 0.042	0.73 ± 0.005	0.94 ± 0.001
*Helcaraxe*, *I*_obs_	39/40	145/157	12	1	0.93 ± 0.036	0.98 ± 0.001	0.92 ± 0.001
*Helcaraxe*, *F*_obs_	26/28	122/126	4	2	0.96 ± 0.027	0.93 ± 0.003	0.97 ± 0.001

## Footnotes

1 Helcaraxë Quenya. Elvish word meaning ‘jagged hedge of spikes’; name of the icy wastes that lay north of Middle Earth.
